# An Expert Diagnostic System to Automatically Identify Asthma and Chronic Obstructive Pulmonary Disease in Clinical Settings

**DOI:** 10.1038/s41598-018-30116-2

**Published:** 2018-08-03

**Authors:** Almir Badnjevic, Lejla Gurbeta, Eddie Custovic

**Affiliations:** 1 International Burch University, Faculty of Engineering and Natural Sciences, Genetics and Bioengineering Department, Sarajevo, Bosnia and Herzegovina; 2 Technical faculty University of Bihac, Bihac, Bosnia and Herzegovina; 3 Medical Device Inspection Laboratory Verlab Ltd, Sarajevo, Bosnia and Herzegovina; 4 Faculty of Electrical Engineering University of Sarajevo, Sarajevo, Bosnia and Herzegovina; 5 La Trobe Innovation & amp, Entrepreneurship Foundry at La Trobe University Melbourne, Melbourne, Australia

## Abstract

Respiratory diseases such as asthma and chronic obstructive pulmonary disease (COPD), are affecting a huge percentage of the world’s population with mortality rates exceeding those of lung cancer and breast cancer combined. The major challenge is the number of patients who are incorrectly diagnosed. To address this, we developed an expert diagnostic system that can differentiate among patients with asthma, COPD or a normal lung function based on measurements of lung function and information about patient’s symptoms. To develop accurate classification algorithms, data from 3657 patients were used and then independently verified using data from 1650 patients collected over a period of two years. Our results demonstrate that the expert diagnostic system can correctly identify patients with asthma and COPD with sensitivity of 96.45% and specificity of 98.71%. Additionally, 98.71% of the patients with a normal lung function were correctly classified, which contributed to a 49.23% decrease in demand for conducting additional tests, therefore decreasing financial cost.

## Introduction

CHRONIC obstructive pulmonary disease (COPD) is a chronic inflammatory lung disease that causes obstructed airflow in the lungs^1,2^. Up to 75% of all COPD patients are not diagnosed. Currently, the COPD mortality rate exceeds that of lung cancer and breast cancer combined^3–6^, as 200,000 to 300,000 deaths in Europe alone are COPD related. Similarly, asthma is a chronic inflammatory impairment of airways, which, as a result, becomes hyperactive and generates increased mucus, mucosal swelling and contraction of smooth airway muscles. These factors all contribute to airway obstruction. With respect to other chronic respiratory diseases, asthma has relatively low fatality rate, but the prevalence of asthma as well as the costs of asthma treatment and care has increased in recent decades^1,2^.

A major challenge in chronic disease management, especially in non-specialized clinics, is the number of patients with chronic respiratory diseases, such as asthma or COPD, who are either inaccurately diagnosed or misdiagnosed for having other respiratory diseases such as the common cold, acute bronchitis or pneumonia^6,7^. Over the years, various evidence-based guidelines for the prevention, diagnosis and management of chronic respiratory diseases have been developed to assist medical professionals. The Global Initiative for Chronic Obstructive Lung Disease (GOLD)^8^ and Global Initiative for Asthma (GINA)^9^ has published guidelines for medical professionals based on their latest research and recommendations. Despite the availability of these guidelines, the lack of knowledge among non-specialized medical professionals is still a leading issue in the correct diagnosis of these respiratory diseases^10^. In 2008, Yawn and Wollan^10^ showed that many primary medical professionals are unable to distinguish asthma from COPD and do not recognize that women are at a higher risk for COPD than men. When COPD symptoms are misdiagnosed as asthma, women receive the wrong treatment, and the correct COPD treatment is delayed. This misdiagnosis has serious adverse consequences in terms of disease burden and risk of future exacerbations.

The usage of computer-based methods in medical diagnoses are on the rise and are gradually improving the quality of medical services by utilizing larger datasets of symptoms and patient history, as well as diagnostic test results for diagnosis. Artificial intelligence and machine learning methods are being used to create expert systems that can employ human knowledge and solve problems that ordinarily require direct human expertise^11,12^. Beginning in the 1990s and increasing in the 2000s, expert systems based on machine learning methods, such as artificial neural networks (ANNs) and fuzzy logic (FL) were used for the detection of different types of diseases, including respiratory diseases. Walia *et al*.^13^ presented a systematic approach for design and identification of tuberculosis using a fuzzy based decision support system. Asaithambi *et al*.^14^ classified respiratory abnormalities using an adaptive ANN and FL inference system based only on spirometry (SPIR) or impulse oscillometry measurements (IOS). Stavrakoudis *et al*.^15^ separated lung sounds, obtained from patients with pulmonary pathology using a recurrent neuro-fuzzy filter. Several studies focusing on the use of different types of ANN architectures for classification of respiratory diseases with high classification accuracies developed on various datasets have been undertaken^16–20^. Other machine learning techniques, such as Random Forests, Gradient Boosting, or even Logistic Regression can also be used for prediction and disease classification^17–19^, but neural networks show optimal performance for this task when there is larger number of samples in databases. However, earlier studies based their classification efforts predominantly on SPIR and/or IOS measured test results, i.e. static assessment of patients. Even though high classification accuracies were reported, additional full dynamic assessments of patients are needed to acquire knowledge about a patient’s symptoms and pulmonary function. Measurement of lung function is important because patients often do not recognize mild symptoms or do not attach importance to them, especially if they persist for a longer period of time. Furthermore, no previously developed system was tested in real-time in clinical settings, that is, all systems were developed and validated offline.

The purpose of this study was to evaluate the impact of introducing Expert Diagnostic System (EDS) into a healthcare system. Our hypothesis was that an accurate EDS could differentiate patients with asthma, COPD and a normal lung function with a classification rate of over 90%. To test our hypothesis, we developed an EDS based on data from 3657 patients and utilized combined ANN and FL algorithms. We then implemented the developed system in a local hospital in Sarajevo, Bosnia and Herzegovina. The EDS system was tested on 1650 patients, and our results showed that our system could achieve a classification rate well above 90%. Our results demonstrate that the developed EDS system is reliable and such an automated diagnosis tool would be beneficial for healthcare institutions, especially in primary care and remote healthcare institutions.

## Materials and Methods

### Implemented expert diagnostic system

**Figure 1:**
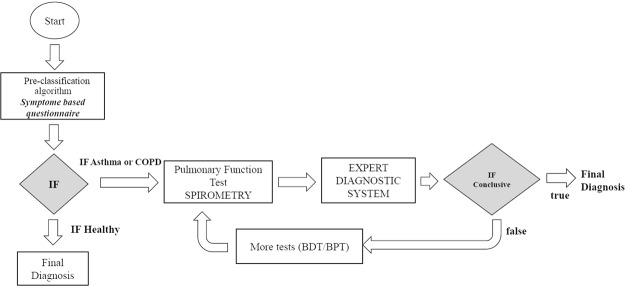
A block diagram of the entire expert diagnostic system.

A block diagram of the EDS for automated identification of COPD and asthma is presented in Fig. 1. It consists of (1) a pre-classification algorithm used to determine whether confirmatory respiratory function tests are needed based on a symptom questionnaire and (2) a classifier based on a combination of a single-layer ANN and FL.

The first step of this EDS is to utilize a pre-classification algorithm to perform initial screening of all patients (healthy vs. COPD/asthma) based on answers provided to a symptom-based questionnaire. From the patient’s answers, the probability of having COPD or asthma is determined. If the calculated probability indicates that the patient falls into the “Normal” category, the patient is diagnosed as “Healthy” and the process ends. However, if the pre-classification algorithm indicates that the probability of being affected by asthma or COPD is greater than 50%, SPIR tests need to be performed. The results of the pulmonary function test are then used as inputs for the implemented Expert Diagnostic System (EDS). The EDS determines whether a conclusive diagnosis, based on spirometry measurements, can be derived (i.e. resulted in a final diagnosis) or further tests (such as bronchodilatation testing- BDT and/or broncho-provocation testing - BPT) are required to reach a more robust decision. Once all required tests are analyzed by the EDS, the final decision is obtained.

### Pre-classification algorithm

To determine the need of running the EDS and performing confirmatory respiratory tests on patients, a pre-classification algorithm was developed. Inputs to the pre-classification algorithms are patient’s answers on seven “yes” or “no” questions about symptoms as shown in Table 1. Based on these answers, the algorithm then calculates the probability of the patient being healthy or diseased (asthma, COPD). These calculations make it possible to identify patients that are required to take a confirmatory test for diagnosis and determine the most appropriate test for the patient. The seven questions, shown in Table 1, were formed in accordance to GINA and GOLD guidelines and took into consideration factors such as age, history of exposure to tobacco and/or other smokes at an age over 40, history of progressive, exertion or persistent dyspnea, occupational dusts, chronic intermittent or non-productive cough and chronic sputum.

**Table 1:** questionnaire form and significance factors for each question related to asthma and copd.

No.	Question	Answer	Asthma (As)	COPD (C)
Q_1_	Does the patient exceed the age of 40 years?	If answered affirmatively the patient **may** have **COPD**. If the answer is negative the patient **may have asthma**.	sf_1As_	sf_1C_
Q_2_	Does the patient experience problems while exercising or performing low intensity activities?	If answered affirmatively the patient **may** have **COPD**.	sf_2As_	sf_2C_
Q_3_	Does the patient cough at night or right after waking up?	If answered affirmatively the patient **may** have **asthma** or **heart disease**.	sf_3As_	sf_3C_
Q_4_	Does the patient suffer from an abundant presence of mucus in the throat?	If answered affirmatively the patient **may** have **COPD**.	sf_4As_	sf_4C_
Q_5_	Does the patient experience high-pitched breath sounds during the morning?	If answered affirmatively the patient **may** have **COPD**.	sf_5As_	sf_5C_
Q_6_	Does the patient experience high pitched breath sounds at night, or while working out, or while performing low intensity activities?	If answered affirmatively the patient **may** have **asthma**.	sf_6As_	sf_6C_
Q_7_	Does the patient experience a choking sensation, while at rest?	If answered affirmatively the patient **may** have **COPD** or **asthma**.	sf_7As_	sf_7C_

*sf_*jk*_ *for the j*^*th*^ *question and k*^*th*^ *disease* Given the abovementioned parameters, the probability (p_k_ for the kth disease) of the presence of asthma or COPD in a patient can be calculated using the following equation.

To quantify the answers given by patients, each answer from the questionnaire was given an option factor (*O*_j_ for the *j*^th^ question). A binary value was assigned to *O*_j_: with a positive answer assigned as 1 and a negative answer as 0. To calculate the probability of disease, a weight was assigned to each answer according to Table 1. These weights were determined in accordance to GINA and GOLD guidelines since not all symptoms are equally significant in the diagnosis of COPD or asthma.

To calculate the probability of disease, a constant of normalization (A_k_ for the kth disease) for asthma and COPD was calculated as the inverse of the sum of the factors:

1 $${A}_{k}=\frac{1}{{\sum }_{i=1}^{i=N}s{f}_{jk}}$$

2 $${p}_{k}={A}_{k}\times \sum _{i=1}^{i=N}(s{f}_{jk}\times {O}_{j})\,\times 100( \% )$$

The probabilities obtained in this process represent the certainty that a patient has indicated type of respiratory disease (asthma or COPD).

Once the patient was pre-classified, it was determined whether a confirmatory test was necessary and, if so, which test was to be performed. The information gathered from these tests was later used as input data for the Expert System. Diagnoses, established by medical professionals, were used to evaluate the pre-classification algorithm and improve it. Both the error (Ek for the kth disease) and the accuracy of the pre-classification could be obtained once these variables were known by using equations (3) and (4).

3 $${E}_{k}={p}_{k}^{c}-{p}_{k}$$

where pk is the diagnosis performed by the medical professional.

4 $$Accuracy=\,\frac{{p}_{k}}{{p}_{k}^{c}}\times 100( \% )$$

### The expert diagnostic system (ANN-FL logic)

In this paper, a feedforward neural network was used for the classification of COPD and asthma since it is proven that this type of neural network architecture is sufficient for these tasks^21–25^. The results from the pulmonary function test, parameters: VC (Vital Capacity), FEV1 (Forced Expiratory Volume in the 1^st^ second), FVC (Forced Vital Capacity) and FEV1% (Tiffeneau index), were introduced as inputs in the ANN, resulting in 4 neurons in the input layer. The fifth input is the result of the pre-classification algorithm, which represents the probability of disease. To standardize the range of independent variables all inputs to neural network were normalized using min-max normalization. This neural network architecture has one output neuron that is the index of 10 output classes being as follows: ASTHMA1, ASTHMA2, ASTHMA3, ASTHMA4, GOLD1, GOLD2, GOLD3, GOLD4, NORMAL and INCONCLUSIVE.

Each neuron in the hidden layer performs a weighted summation of the inputs, which is then passed to a non-linear activation function. The hidden layer neurons use nonlinear hyperbolic tangent sigmoid transfer function, while the linear activation function was used for the output layer. Although this function is commonly used in regression problems^26^, the system with these transfer functions in each layer showed optimum performance. A weight matrix (Iws, where s = 1…S (input neurons) and n = 1…N (hidden neurons)) was automatically defined to the links connecting input and hidden neurons (Hn) following the training algorithm. An activation function (HOn) was computed for each hidden neuron, taking into account its bias (Hbn):

At this point, a new weight matrix (Hw_n, r_ where n = 1…N (hidden neurons) and r = 1…R (output neurons)) was assigned to the links, connecting the neurons in the hidden and the output layer (Or). The outputs (yr) were then calculated by considering the abovementioned parameters and the corresponding bias in each output neuron (Obr):

The number of neurons in the hidden layer was determined by evaluating the performance of the neural network architectures with 5, 10, 14 and 20 neurons. The tested neural network architectures were compared based on accuracy of classification of COPD and asthma.

The fuzzy logic output classifier was developed based on recommendations by experts in this field^25–30^ and international guidelines, GINA and GOLD. Input variables for the implemented fuzzy logic output classifier are the result of BDT/BPT tests and information about the disease probability. The fuzzy logic rules were defined based on the severity of the disease, asthma or COPD and information about disease probability. Based on the fuzzy rules, outputs for the diagnosis were defined as COPD, asthma or healthy^20^.

### Algorithm training and testing

During the development of a neural network, instead of dividing the dataset into distinct groups for training, validation and testing with a fixed number of samples, k-fold cross validation was used. The overall dataset for the development of the neural network was divided into *k* subsets. At each learning iteration, the neural network was trained on k −1 subsets, then tested on the one subset, which was not used during training. This process is repeated k times, each time using a different test set chosen from the k available divisions of the training data, until all possible test sets have been used. The k test set performances for each model are averaged, and the model with the highest average performance is chosen as the one most likely to perform well on unseen data. For training, the Levenberg – Marquardt algorithm (LMA), which is a common training algorithm in data classification was used^29^. As a measure of performance, at each training iteration, the Mean Square Error (MSE) between the predicted and actual values was calculated, where n is total number of samples:

5 $$MSE=\frac{1}{n}\sum _{i=1}^{n}{({X}_{predicted}-{X}_{actual})}^{2}$$

The classification accuracy in relation to the number of neurons in the hidden layer was also examined. While the number of input and output neurons was determined with data structure and process modeling, the performance of training was dependent on the complexity of the neural network and the number of neurons in the hidden layer^31^. A poorly defined number of neurons in the hidden layer can result in overfitting the problem. This overfitting leads to very high training performance (accuracy of classification >90%) and very poor testing performance (accuracy of classification <60%). There are various methods for choosing the fixed number of neurons in the hidden layer, but there is no generally accepted method for determining the number of neurons in a single hidden layer that would efficiently approximate any given function or process. Despite the new methods developed for this purpose, most researchers use a trial rule^25^. This rule was used in this study as well. The number of neurons in the hidden layer was set to be 5, 12, 14, 17 and 20 and their performance was calculated. The smallest MSE was achieved with 17 neurons in the hidden layer (12.485), but since the neural network with 14 neurons in the hidden layer (12.569) had a marginally poorer performance, 14 neurons were chosen for further development to avoid a more complex architecture.

### Sample collection

Following internationally accepted medical practices for diagnosis of COPD and asthma, a prospective study was developed to generate the dataset for design, validation and real-time use of EDS, for automated identification of asthma and COPD. Before starting the study, the ethics board approval for human subject testing from the Hospital Sarajevo was obtained, as well as the patients’ informed consent. Healthcare institutions also approved all methods and procedures which were performed in accordance with the relevant guidelines and regulations. Finally, a dataset of 3657 samples based on patient reports was generated, which included relevant information established by medical professionals. Out of the 3657 samples, 3000 previously collected 2014 and 2015 samples were used for development and training of ANN, while 657 samples were used for validation of fuzzy classifier in EDS.

The whole implemented EDS was tested with 1650 samples in real-time at the Pulmonary Clinic of Sarajevo and primary healthcare institutions during 2016. The class distribution of the dataset is presented in Table 2. In the dataset of 1650 samples, 1495 samples were samples of diseased subjects (859 of asthma and 636 of COPD) and 155 were samples of healthy subjects. During the real-time clinical testing of the EDS, parallel diagnoses were performed by medical professional and the EDS. The EDS diagnosis was revealed to medical professionals after they formalized the diagnosis for the same patient. At the end, the EDS diagnosis was finally compared with the medical professional diagnosis.

**Table 2:** Dataset Distribution per Classes.

	Asthma		COPD	Healthy	
Total number of samples	**5307**		**2485**	**1514**	**1308**
Samples used for ANN development *2014–2015	3000 (56.53%)		1267 (50.98%)	689 (45.51%)	1044 (79.82%)
Samples used for fuzzy classifier development *2014–2015	657 (12.38%)		359 (14.45%)	189 (12.48%)	109 (8.33%)
Real – time testing in Pulmonary Clinic of Sarajevo *during 2016	1650 (31.09%)		859 (34.57%)	636 (42.01%)	155 (11.85%)
*Etymology of real – time testing data					
*Sex*	*896 Male*			*754 Female*	
*Age (Average)*	*44*.*12*			*49*.*23*	
*Symptoms*	*72*.*3% of subjects have 3 or more symptoms of disease*			*81*.*6% of subjects have 3 or more symptoms of disease*	
*Other diseases diagnosed*	*15*.*7%*			*13*.*1%*	
*Consummation of medications during past 7 days*	*54*.*2%*			*49*.*7%*	

Each sample contained seven features representing the patient symptoms information and four features representing the results of the SPIR tests. Additionally, some samples had features representing the results of BDT/BPT test in the case that it was undertaken. Only patients with confirmed a diagnosis were subject to this study. Diagnoses were performed by medical professionals following clinical assessment according to international guidelines^8,9^. Baseline assessments consisted of screening for patient symptoms using symptom based questionaries’ or interview conducted by a medical professional. All spirometry lung function tests were obtained using the CareFusion “Master Screen” device, which measured, derived and calculated all the required spirometry parameters that were features of samples.

The etymology of the data (patient age, sex, treatment) used for development and training of EDS was not analyzed since they are part of already established disease diagnosis, while etymology of data from real-time testing of EDS was analyzed and presented in Table 2.

## Results

The pre-classification algorithm was validated on 2735 samples from the overall dataset. During validation of the algorithm, the accuracy of 95.17% for samples with COPD or asthma and 98.7% for pre-classification of healthy subjects were both achieved.

It should be noted that the results of the pre-classification algorithm are also checked (confirmed or discarded) by medical professional so the quality of patient care is not decreased. The rate of misdiagnosis by pre-classification algorithm is still lower than currently available information on COPD and asthma diagnosis.

By introducing computer-aided systems with pre-classification algorithms in everyday practice, very high savings on respiratory function tests can be achieved and the quality of care is not affected. In particular, savings of 98.7% of total SPIR costs (see Table 3) can be achieved on 1291 patients that were correctly classified as healthy patients. When calculating these savings, the average cost of an hourly visit to a medical specialist as well as the costs of blood gas analysis and necessary filters for SPIR tests were taken into account.

**Table 3:** Pre-classification validation accuracy and cost analysis.

No. of reports		True pre-classifications	False pre-classifications	% of true pre-classifications
Disease	2735	2603	132	95.17
**Healthy**	**1308**	**17**	**1291**	**98**.**70**
			**Average:**	**96**.**93**
COPD	1028	959	69	93.33
Asthma	1707	1644	63	96.31
**Healthy**	**No**. **of samples**		Cost of SPIR	
	**1308**			
True pre-classifications	**1291**		**98**.**7%** of SPIR cost for 1308 patients	
False pre-classifications	**17**		**1**.**3%** of SPIR cost for 1308 patients	

*Cost of SPIR testing for healthy patients $60* + *the average price of a filter needed during pulmonary tests $1* + *average costs of hourly visit to medical professional $100 roughly* + *additional blood gas analysis (complete panel price is about $10 per patient)*

*The costs of SPIR testing for Bosnia and Herzegovina are taken as approximate values based on various pricing from public and private healthcare institutions in June, 2017.

The overall EDS was tested in real-time on 1650 consecutive patients enrolled at healthcare institutions in Bosnia and Herzegovina during 2016. The performance of EDS is reported in Table 4.

**Table 4:** System Performance.

	EDS output				
	Number of reports ${\sum }^{}1650$	Disease (COPD & Asthma)	Healthy	Prevalence 90.61%	
True condition	Disease (COPD & Asthma) ${\sum }^{}1495\,$(859 asthma/636 COPD)	1442	53	True positive rate Sensitivity 96.45%	False negative rate Miss rate 3.55%
	Healthy ${\sum }^{}155$	2	153	False positive rate 1.29%	True negative rate Specificity 98.71%
	Accuracy 96.66%	Positive prediction value 99.86%	False condition rate 25.73%	Positive likelihood ratio 74.76%	
		False discovery rate 0.139%	Negative predictive rate 74.27%	Negative likelihood ratio 1.34%	

Out of 1495 patients with some respiratory disease, 1442 were correctly classified resulting in sensitivity of 96.45%. Specifically, out of 859 patients with a confirmed diagnosis of asthma, 96.62% were correctly classified, while, out of the 636 COPD patients, 96.22% were correctly classified by the EDS. In addition, 98.71% of the 155 patients with normal lung function were correctly classified.

While its installment at healthcare institutions in Bosnia and Herzegovina from January to October 2016, implemented EDS results indicated high potential for use in everyday clinical practice. Within the 1495 samples with disease diagnosis, the EDS diagnosed 1123 reports without performing additional BDT/BPT tests, and when compared to diagnosis performed by medical professionals, there was a 49.23% decrease in conducting BPT/BDT tests. Furthermore, the pre-classification algorithm accuracy rate was very high, indicating that in 98.71% cases of COPD and asthma diagnosis performed by medical professionals, confirmatory pulmonary tests could have been avoided.

**Figure 2:**
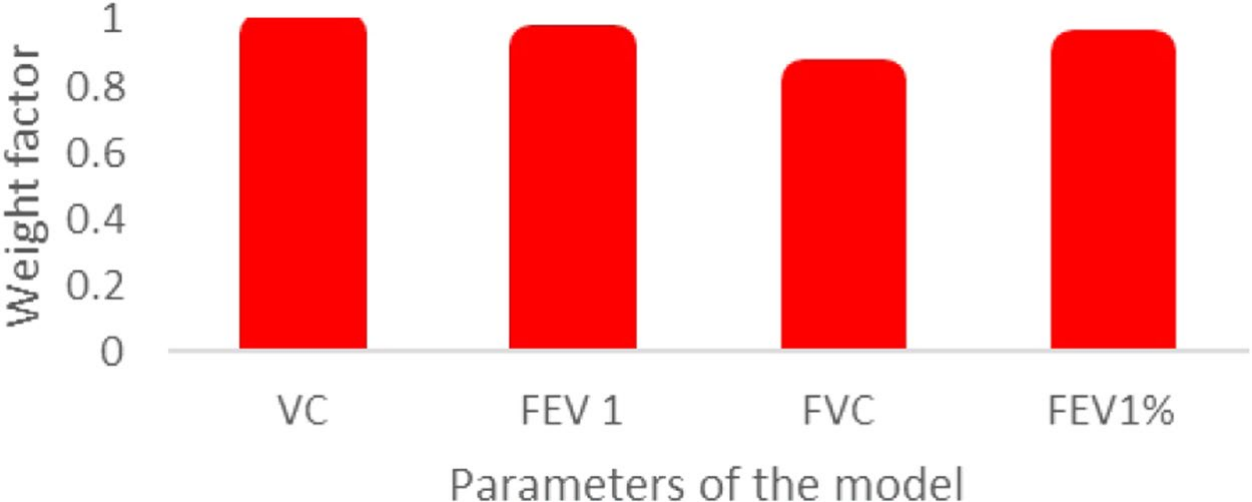
Weight factors of input parameters to machine learning models.

Based on reviewer’s recommendations, after real-time validation of the system performance in healthcare institutions, several models based on Random Forests, Gradient Boosting and Logistic Regressions algorithms^17–19,32–41^ were developed to evaluate results of Expert System relative to these machine learning algorithms. These models were developed based on 3000 samples. Machine learning models had 4 input parameters that were also used as inputs to developed neural network architecture. Weight factors of these inputs to machine learning are presented in Fig. 2.

As it can be seen from Table 5, Gradient Boosting algorithm had the best accuracy in respect to other machine learning models and developed artificial neural network. This is because Gradient Boosting finds best features and focus weight increase on misclassified data so that their importance increases and therefore entire system accuracy.

**Table 5:** Comparison Of Efficency Of Machine Learning Algorithms For Classification Of Asthma And Copd.

Samples ${\sum }^{}3000$	Model types			
	Random Forests	Gradient Boosting	Logistic Regression	Artificial Neural Network
Accuracy	97.330%	98.33%	95.33%	93.60%

Following results from the Table 5, the future research in this area should be continued in direction of usage of machine learning techniques, and not just neural networks.

## Discussion

Due to a population lifestyle that includes smoking and air pollution, as well as other various factors, we have faced challenges for decades in diagnosis and management of various chronic respiratory diseases. Standardized procedures for respiratory disease diagnosis and management are available^8,9^ but these procedures still have not yielded results that significantly reduce mortality rates, especially in remote areas where medical specialists are not always available. Thus, new innovative approaches are needed to help cope with this kind of disease. Considering the success of artificial intelligence and fuzzy logic had in other scientific disciplines, it was anticipated to yield similar results when applied to the problem of respiratory disease diagnosis, especially COPD and asthma. Indeed, our results show that the automated approach in disease diagnosis provides a useful solution yielding in an increase of timely correct diagnosis, especially for non-specialized medical professionals in remote areas. Based on this, early intervention programs can be developed to prevent disease complications and to ensure patients have timely delivered care.

There have been several studies focusing on diagnosing respiratory diseases using various types of artificial neural network architectures and fuzzy logic classifiers, as well as expert systems structures based on both methods. These studies have applied different neural network structures for various respiratory diseases diagnosis, including asthma and COPD, using datasets with various features^42–51^. Consistent with previous research in this area^13–20^, an automated system for COPD and asthma diagnosis based on fuzzy logic and artificial neural network was developed. Databases with previously established and confirmed diagnoses were generated for system development and patients were divided into three, mutually-exclusive categories: (1) healthy, (2) COPD, and (3) asthma. This database consisted of parameters describing patient etiology and physical examinations. During the data collection, it was noticed that patients who lived in more urban areas were more likely to have a diagnosis established earlier than those who lived in rural areas and had to go through more levels of healthcare. This means that patients from rural areas are at a higher risk of having complications and inadequate care. so Therefore, an important intervention would be to have the early diagnosis of disease at primary healthcare units which could be achieved through implementation of an automated EDS such as the one described in this research. The goal of implementing these systems in primary healthcare institutions would be to connect patients with services that would help them obtain timely care for their health and improve their access to healthcare resources.

The diversity of variables identified in the proposed EDS illustrate that medical professionals should not make a diagnosis based only on results from biological or chemical tests using laboratory values or radiological images. The patient should be evaluated as a whole system, taking into consideration functional testing factors such social factors, habits, nutrition, and epidemiological factors, as prescribed in international guidelines^8,9^. In this research, a total of eleven different parameters were used while performing diagnosis, with an additional five resulting from testing if needed. Patient symptoms are taken with different significance factor in the pre-classification algorithm which corresponds to international guidelines and long-term medical specialist experience. Due to the availability of spirometers and their cost, spirometry was chosen for functional testing during this research which is also in accordance with the aforementioned international guidelines, GINA and GOLD.

In assessing the strengths and weaknesses of this study, one of its major strengths is the high true positive rate of 97.58% suggesting the high EDS accuracy. As expected, artificial intelligence and fuzzy classifiers yielded high accuracy when implemented on respiratory disease. To place this result into perspective, similar performances have been achieved in other contributions^32–36^, but our proposed system was evaluated on both a larger dataset and real clinical settings. Furthermore, the EDS has proven reliability of results since all diagnosis used for training were previously confirmed by trained medical specialist. This system also takes into account other patient symptoms beside the results from the functional lung test, all of which have been found to be highly associated with COPD and asthma. Finally, the real-time testing proved the utility of this system, as a simple graphical user interface system satisfied medical professional’s needs.

Medical professionals included in the study had a very good response to the application. They were intrigued by the number of parameters that could be simultaneously analyzed and the time needed to generate a diagnosis. Most of medical professionals included in real-time evaluation of the system commented that it was a useful tool which helped them support the decision of patient diagnosis. Also, they all agreed that it helps reduce costs of unnecessary testing. Nevertheless, all diagnoses were finally made by the medical specialist following the recommended procedure for each patient, so false positives were treated accordingly. They undertook tests and the final diagnosis was established regardless of the system output.

As with all research, our study does have some limitations. This database consists only data collected in Bosnia and Herzegovina. It would be useful to test the system performance on data collected in different populations to evaluate the system’s sensitivity. Furthermore, real time evaluation of the developed EDS was performed in only one healthcare institution in Bosnia and Herzegovina. Therefore, to further validate this automated EDS we recommend testing in other healthcare institutions.

Automated expert diagnostic systems are useful in healthcare practice because they enable diagnostics based on larger datasets and are able to take into account multiple input parameters at once. Also, the results performed by these systems are also objective and cannot be influenced by various factors. These systems are especially useful in remote healthcare institutions where a medical specialist, in this case a respiratory medical specialist, is not available and a general practitioner is not fully confident in establishing the patient diagnosis nor determining a future treatment. Accurate diagnosis of respiratory diseases in these institutions is often missed since numerous symptoms are misjudged, leading to a greater percentage of misdiagnosis or late diagnosis. These errors lead to significant negative effects on patients’ health overall. The proposed EDS offers the opportunity for medical professionals to run patient symptoms through a large database of knowledge and calculate the probability of a patient having the disease. Timely diagnosis is very important step in disease treatment so in these cases, the result of EDS helps medical professional in the prescription of medication or recommendations for additional confirmatory testing. This in turn, leads to the reduction of mortality rates and the improvement of patient quality of life. It should be noted that the usage of these systems does not implicate that medical professionals are a replaceable factor in establishing diagnosis. On the contrary, these systems assist medical professionals in providing quality treatment for patients.

In the future, the authors plan to improve and validate an effective user interface for the automated EDS, enabling the usage of this system in general practitioners’ offices in healthcare institutions. This system would assist general practitioners in performing preliminary diagnoses leading to optimization of time resources, decreased medical device associated costs and enhanced patient outcomes.

## Conclusion

This paper presented a novel EDS for COPD and asthma diagnosis. During development of the EDS, the system was trained with over 3000 reports acquired from 2014 to 2015 in a pulmonary clinic using the CareFusion device. Subsequently, the EDS was validated using new data acquired in a prospective study conducted at a local healthcare institution. The developed system correctly classified over 97% of the 1650 enrolled subjects, achieving a sensitivity of over 96% and specificity over of 98%.
